# Global Trends and Hotspots in Research on Tooth Agenesis: A 20-Year Bibliometric Analysis

**DOI:** 10.7759/cureus.46961

**Published:** 2023-10-13

**Authors:** Bo Xie, Ying Han, Xiujie Wen

**Affiliations:** 1 Department of Orthodontics, Affiliated Stomatological Hospital of Southwest Medical University, Luzhou, CHN; 2 Department of Oral and Maxillofacial Surgery, Center of Stomatology, Xiangya Hospital, Central South University, Changsha, CHN

**Keywords:** tooth agenesis, oligodontia, hypodontia, bibliometric analysis, anodontia

## Abstract

Tooth agenesis, one of the most common developmental defects in humans, not only impairs oral function but can also lead to craniofacial deformities. Bibliometric analysis can reveal significant shifts in research and publishing trends within specific fields. This study aims to provide a comprehensive overview of the research hotspots in tooth agenesis and predict future trends through bibliometric analysis. We searched for English-language publications related to tooth agenesis from 2001 to 2021 on the Web of Science. The publications were limited to original and review articles, and bibliometric parameters such as publication year, country, institution, author, journal, citations, and keywords were extracted and analyzed using VOSviewer, Microsoft Excel 2010, and CiteSpace. A total of 2,287 papers were ultimately selected. The results show that the USA holds a leading position in the field of tooth agenesis research. A total of 9,803 authors participated in these studies, with Alexandre R Vieira from the USA being the most prolific and most cited author. This study indicates that multidisciplinary management has become the consensus first choice for treating dental agenesis. Gene mutations related to tooth agenesis continue to be a research hotspot attracting scholarly attention. Exploring the relationship between tooth agenesis and cancer may be a future research direction. These findings contribute to potential collaborations among experts in future research on the genetic causes of tooth agenesis and tumor development and to assist the scientific community by identifying research gaps in this field.

## Introduction and background

As one of the most common developmental defects of human beings, tooth agenesis has been reported worldwide. The incidence of tooth agenesis is 2.2-10.1%, which varies according to different populations [1]. Based on the number of missing teeth, excluding the third molar, tooth agenesis is classified as hypodontia (missing one to five permanent teeth), oligodontia (missing six or more permanent teeth), or anodontia (completely missing teeth) [2]. Tooth agenesis can have adverse physiological and psychological effects on patients. On the one hand, tooth agenesis causes serious damage to the oral chewing function of patients, decreasing chewing efficiency and reducing patients’ quality of life; on the other hand, articulation disorder and facial deformity problems caused by tooth agenesis seriously affect patients’ mental health [3].

Tooth agenesis can occur not only as an isolated condition but also as part of a syndrome [4]. Msh homeobox 1 (*MSX1*) and paired box protein 9 (*PAX9*) were discovered as the first and second genes associated with tooth agenesis [5,6]. Subsequently, axis inhibition protein 2 (*AXIN2*), ectodysplasin A (*EDA*), and other genes were also found to cause dental agenesis [7]. In addition, various complex diseases are associated with tooth agenesis, such as X-linked hypohidrotic ectodermal dysplasia (XLHED) [8].

Given the significant challenge that tooth agenesis presents to global oral health, scholars have conducted numerous reviews on its pathogenesis, clinical manifestations, and treatment strategies. However, certain shortcomings need to be addressed, such as the subjective selection of samples in some systematic reviews and the small sample sizes. Moreover, these reviews do not comprehensively include all studies, thus lacking a thorough quantitative analysis of research on tooth agenesis. Bibliometric analysis is a research method that provides qualitative and quantitative analysis of existing publications in a specific field [9]. Analyzing the number of publications, citations, authors, institutions, and keywords can reflect the global trends and hotspots in the field. This study aims to conduct a comprehensive bibliometric analysis of the hotspots and trends in clinical and basic research on congenital tooth agenesis and to assess its impact. This will provide direction for subsequent research aimed at addressing the global challenge of tooth agenesis.

## Review

Bibliometric methodology

Search Strategy

We searched the Web of Science Core Collection (WoSCC) using the following items: (TS = tooth agenesis OR dental agenesis OR hypodontia OR anodontia OR oligodontia OR congenital missing teeth) from January 1, 2001, to January 1, 2021. The language was restricted to English. Two investigators independently searched and screened the database on November 15, 2021. Disagreements were resolved by discussion with a senior dentist until a consensus was reached.

Data Extraction and Analysis

The title, author, institution, country, publication year, keywords, and citations were listed as extracted information for bibliometric analysis. Microsoft Excel 2010 (Microsoft Corp., Redmond, WA, USA) was used to count the contributing authors, journals, institutions, and countries. Coauthor-authorship, coauthor-institution, coauthor-country, and keywords were further visualized using VOSviewer (Leiden University, Leiden, the Netherlands). In order to detect any abrupt change in the frequency of references and keywords, CiteSpace (Version 5.8. R1) was applied to burst detection.

Results

**Figure 1:**
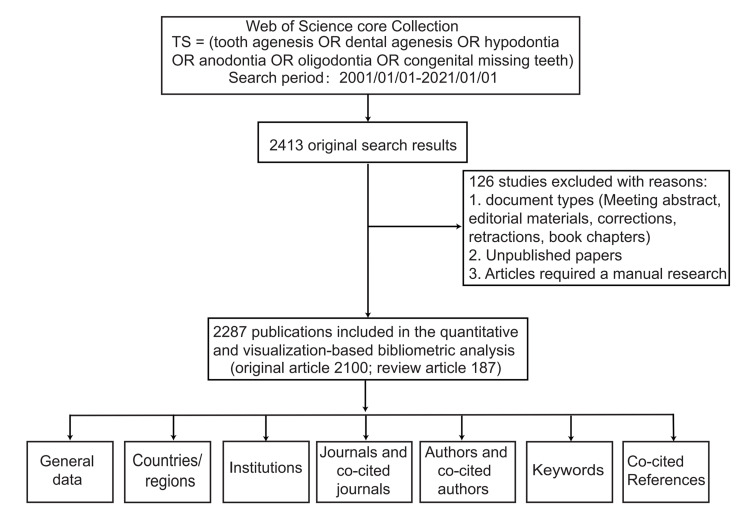
Flowchart of data screening and bibliometric analysis.

**Figure 2:**
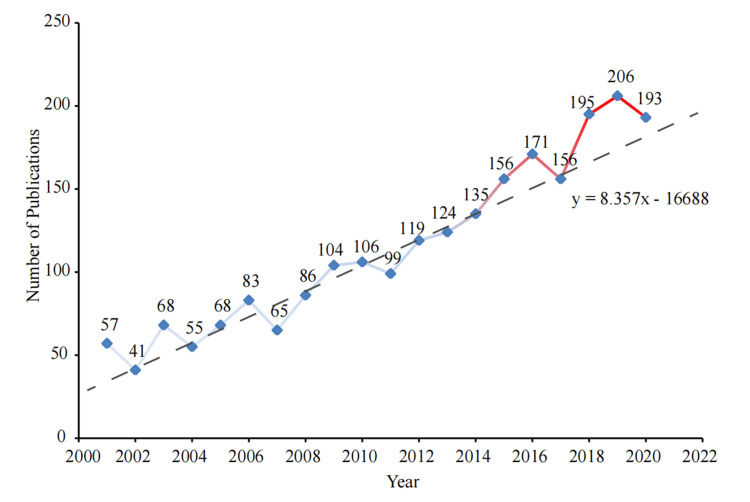
Number of publications on tooth agenesis per year from 2001 to 2021.

After excluding non-English papers and restricting to original and review articles, 2,287 publications were finally selected for further bibliometric analysis (Figure 1). Of the 2,287 articles, 2,100 were original articles (91.8%), and 187 were reviews (8.2%). The annual publication outputs showed an overall upward trend (Figure 2).

Countries/Regions and Institutions

**Figure 3:**
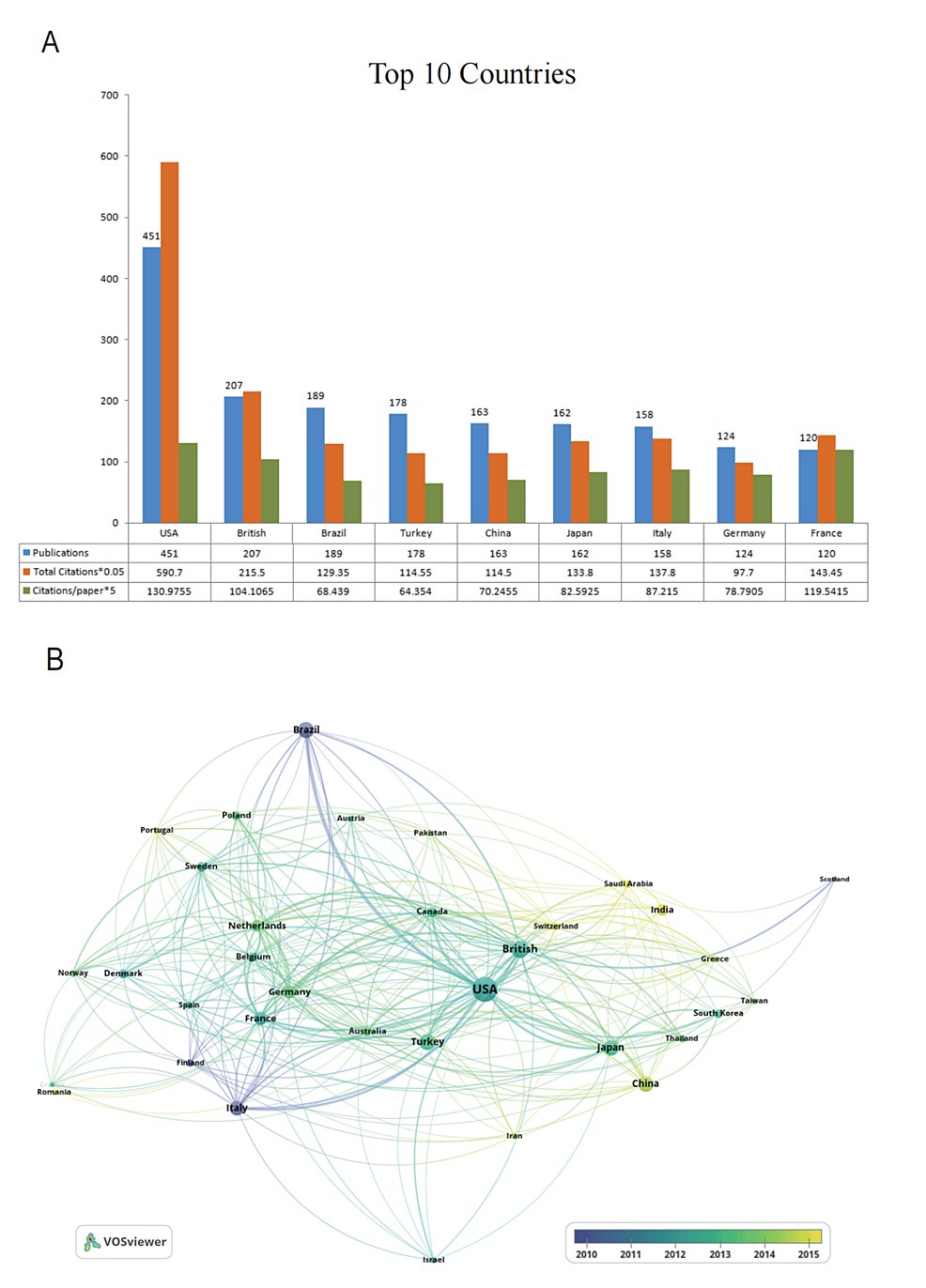
Contributions of top 10 countries and international collaborative networks to tooth agenesis-related research. (A) The number of publications and total citations per country. (B) Cooperation between countries. A larger node represents a higher number of articles, and a wider line between nodes indicates greater strength cooperation.

**Figure 4:**
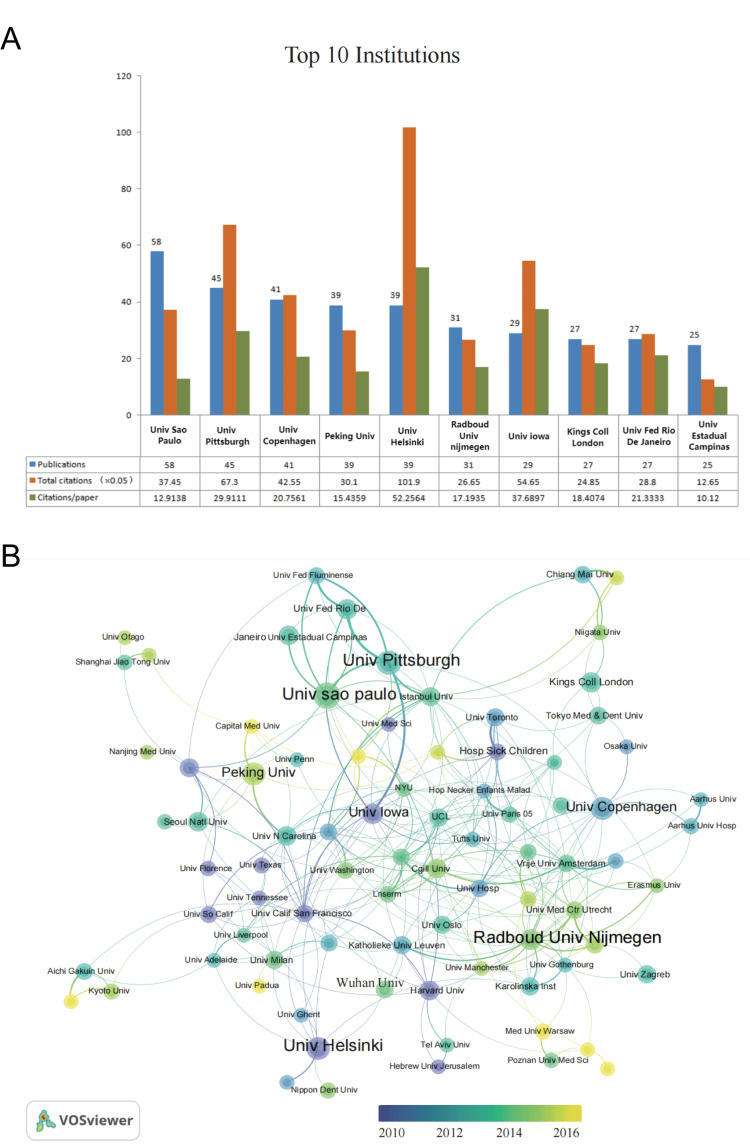
Distribution of institutions focusing on tooth agenesis and cooperative relationship between institutions. (A) Publications and total citations for top 10 prolific institutions. (B) The international collaboration visualization map of institutions. The size of the node and thickness of connection lines represent the number of published articles and cooperative strength, respectively.

Figure 3A shows the top 10 prolific countries/regions. The USA ranked first in this field, more than double that of Britain and Brazil, which ranked second and third. We analyzed the collaborations of countries/regions using VOSviewer (Figure 3B). The USA established partnerships with 31 countries, which is the most cooperative with a total link strength of 372. Regarding dynamics and trends, Brazil, Italy, and Finland have focused on this field since 2010. The USA, Britain, Turkey, and France have been active from 2001 to 2021. After 2015, China, India, and Switzerland started playing more active roles in this field. In the top 10 institutions (Figure 4A), the University of Sao Paulo (Brazil) ranked first in the number of publications (n=58). Regarding average citations per paper and total citations, the University of Helsinki ranked first, followed by the University of Pittsburgh and the University of Iowa. The cooperative relationship of these institutions is further visualized in Figure 4B.

Authors

The top 10 most productive authors and co-cited authors may represent influential research teams and potential research partners (Table 1). Alexandre R Vieira (the USA, with 31 publications) was the most prolific author, with the highest total citations (n=590), followed by Dong Han (China, with 20 publications) and Hailan Feng (China, with 19 publications). Co-citation is a significant indicator of the extent an author contributed. The top three most co-cited authors in the field of tooth agenesis research were Brook AH, Polder BJ, and Vieira AR.

**Table 1:** The top 10 prolific authors and co-cited authors on tooth agenesis from 2001-2021.

Rank	Author	Number	Citations	Country	Co-cited author	Co-citations	Country
1	Vieira, Alexandre R	31	590	USA	Brook, Alan H	378	England
2	Han, Dong	20	316	China	Polder, Bart J	369	Netherlands
3	Feng, Hailan	18	346	China	Vieira, Alexandre R	324	USA
4	Letra, Ariadne	12	246	USA	Peck, Sheldon	323	USA
5	Liu, Haochen	11	91	China	Mostowska, Adrianna	317	Poland
6	Bergendal, Birgitta	10	294	Sweden	Nieminen, Pekka	303	Finland
7	Costa, Marcelo De Castro	10	173	Brazil	Ranta, Ronald	271	Brazil
8	D'Souza, Rena N.	10	275	USA	Bergendal, Birgitta	270	Sweden
9	Mues, Gabriele	9	186	USA	Thesleff, Irma	264	Finland
10	Wong, Singwai	9	118	USA	Lammi, Laura	250	Finland

Journals

Table 2 lists the top 10 prolific journals and co-cited journals in descending order. The American Journal of Orthodontics and Dentofacial Orthopedics (n=100 publications) published the highest number of publications related to tooth agenesis, followed by the American Journal of Medical Genetics Part A (n=86 publications) and the Journal of Dental Research (n=73 publications). Regarding co-cited journals, the American Journal of Orthodontics and Dentofacial Orthopedics (3,243 co-citations), American Journal of Medical Genetics Part A (2,209 co-citations), and European Journal of Orthodontics (1,994 co-citations) ranked the first three.

**Table 2:** The top 10 prolific journals and co-cited journals from 2001 to 2021. IF, impact factor

Rank	Journal	Publications	Citations	Citations per paper	IF_2020_	Co-cited journal	Co-citations	IF_2020_
1	Am J Orthod Dentofac	100	1,984	19.84	2.65	Am J Orthod Dentofac	3,243	2.65
2	Am J Med Genet A	86	2,125	24.7093	2.802	J Dent Res	2,209	6.116
3	J Dent Res	73	2,424	33.2055	6.116	Eur J Orthodont	1,994	3.075
4	Cleft Palate-Cran J	69	1,432	20.7536	1.433	Angle Orthod	1,922	2.079
5	Arch Oral Biol	62	933	15.0484	2.633	Nat Genet	1,865	38.33
6	Eur J Orthodont	61	1,477	24.2131	3.075	Am J Hum Genet	1,604	11.025
7	Angle Orthod	52	1,200	23.0769	2.079	Am J Med Genet A	1,393	2.802
8	Brit Dent J	31	523	16.871	1.626	Cleft Palate-Cran J	1,378	1.433
9	Int J Paediatr Dent	31	477	15.3871	3.455	Arch Oral Biol	1,158	2.633
10	Eur J Oral Sci	30	681	22.7	2.61	Am J Med Genet A	1,055	2.802

Cited Articles and Co-cited References

**Figure 5:**
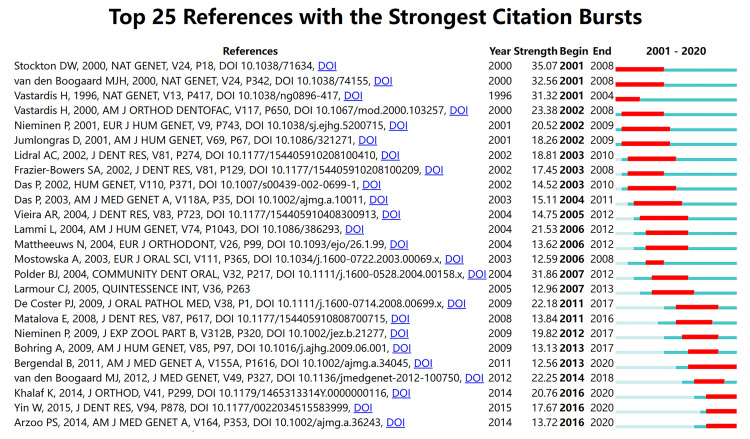
Top 25 references with the strongest citation bursts on tooth agenesis between 2001 and 2021. The red line shows the burst time.

As shown in Table 3, in the top 10 cited articles, there is only one clinical research and three reviews or meta-analyses; the remaining are all basic research. Polder BJ published the most cited article in 2004 in Community Dentistry and Oral Epidemiology with 496 citations, titled “A meta-analysis of the prevalence of dental agenesis of permanent teeth” [1]. This study provides researchers and dentists with applicable data on the prevalence and characterization of dental agenesis from the data of Caucasian populations in three continents. Co-cited references were references co-cited by a set of included papers. Co-cited references help researchers become familiar with dental agenesis quickly. The co-citation network was conducted using 17 references co-cited more than 100 times. Table 4 shows that the article published by Polder BJ was also the most co-cited reference (n=368). The explosive citation of references can reflect the articles researchers pay attention to in a specific period. As shown in Figure 5, “Stockton DW, 2000, NAT GENET, V24, P18, DOI 10.1038/71634” had the highest burst strength, titled “Mutation of *PAX9* is associated with oligodontia” [5]. In this research, the author identified a frameshift mutation in *PAX9* following a genome-wide analysis of a case of family segregating autosomal dominant oligodontia. There are four articles with sharply increased citations at the end of 2020. These articles suggest that researchers are paying attention to the pathogenic genes of dental agenesis, among which the wingless-type MMTV integration site family, member 10A (*WNT10a*), may have new findings.

**Table 3:** The top 10 cited articles on tooth agenesis.

Rank	Title	First author	Journal	Citations	Year
1	A meta-analysis of the prevalence of dental agenesis of permanent teeth	Polder, Bart J	Community Dentistry and Oral Epidemiology	496	2004
2	Mutations in AXIN2 cause familial tooth agenesis and predispose to colorectal cancer	Lammi, Laura	American Journal of Human Genetics	422	2004
3	Rescue of cleft palate in Msx1-deficient mice by transgenic Bmp4 reveals a network of BMP and Shh signaling in the regulation of mammalian palatogenesis	Zhang, Zunyi	Development	296	2002
						4	Hay-Wells syndrome is caused by heterozygous missense mutations in the SAM domain of p63	McGrath, John A	Human Molecular Genetics	225	2006
5	Craniofacial tissue engineering by stem cells	Thesleff, Irma	Journal of Dental Research	217	2006
6	The genetic basis of tooth development and dental defects	Vieira, Alexandre Rezende	American Journal of Medical Genetics Part A	171	2008
7	Fibroblast growth factor receptor 2-IIIb acts upstream of Shh and Fgf4 and is required for limb bud maintenance but not for the induction of Fgf8, Fgf10, Msx1, or Bmp4	Revest, Jean Michel	Developmental Biology	170	2003
						8	Mutations in MTMR13, a new pseudophosphatase homologue of MTMR2 and Sbf1, in two families with an autosomal recessive demyelinating form of Charcot-Marie-Tooth disease associated with early-onset glaucoma	Azzedine, Hamid	American Journal of Human Genetics	160	2001
9	The transcription factor gene Nfib is essential for both lung maturation and brain development	Steele-Perkins, George	Molecular and Cellular Biology	158	2007						
10	Unique functions of Sonic hedgehog signaling during external genitalia development	Haraguchi, Ryuma	Development	154	2001

**Table 4:** The top 10 co-cited references according to total publications from 2001 to 2021.

Rank	Co-cited reference	citations
1	Polder, Bart J, 2004, community dent oral, v32, p217, doi 10.1111/j.1600-0528.2004.00158.x [1]	368
2	Vastardis, Heleni, 1996, nat genet, v13, p417, doi 10.1038/ng0896-417 [6]	281
3	Stockton, David W, 2000, nat genet, v24, p18, doi 10.1038/71634 [5]	248
4	Lammi, Laura, 2004, am j hum genet, v74, p1043, doi 10.1086/386293 [10]	232
5	Vastardis, Heleni, 2000, am j orthod dentofac, v117, p650, doi 10.1067/mod.2000.103257 [11]	186
6	van den Boogaard, M J, 2000, nat genet, v24, p342, doi 10.1038/74155 [12]	171
7	Satokata, Ichiro, 1994, nat genet, v6, p348, doi 10.1038/ng0494-348 [13]	137
8	Kere, Juha, 1996, nat genet, v13, p409, doi 10.1038/ng0895-409 [14]	125
9	Brook, Alan Henry, 1984, arch oral biol, v29, p373, doi 10.1016/0003-9969(84)90163-8 [15]	118
10	De Coster, PJ, 2009, j oral pathol med, v38, p1, doi 10.1111/j.1600-0714.2008.00699.x [16]	118

Keywords

**Figure 6:**
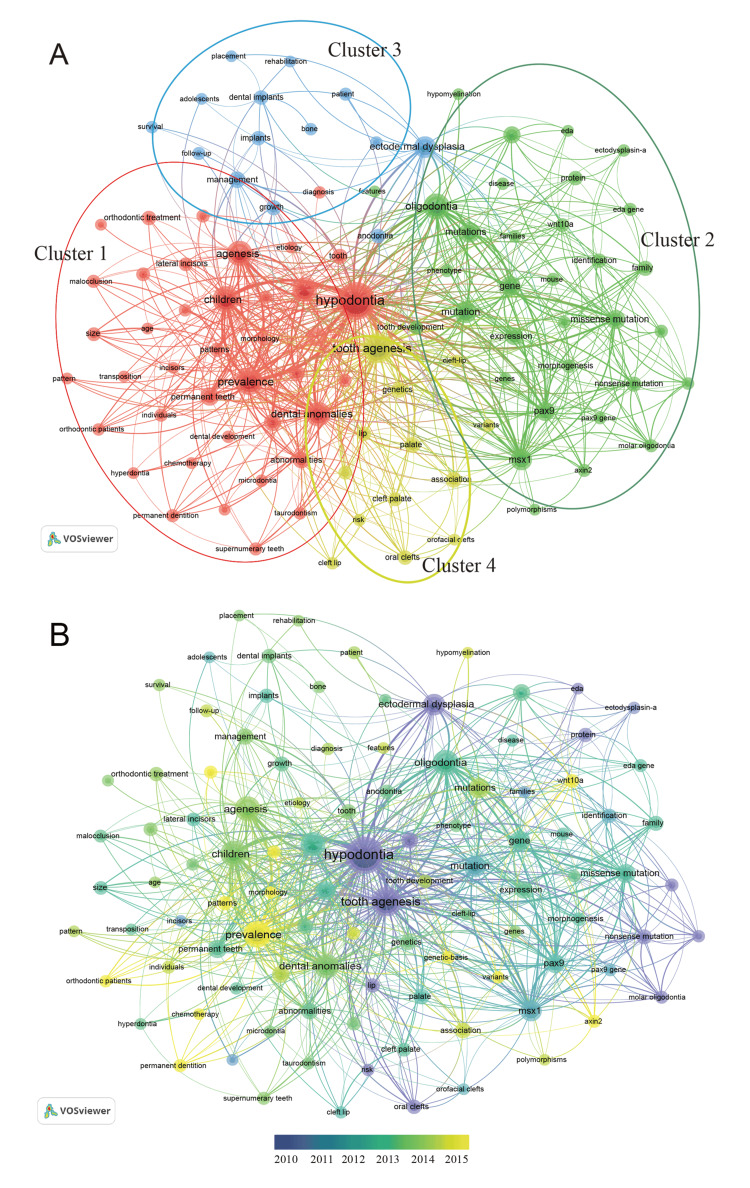
The analysis of keywords in publications on tooth agenesis. (A) The map of keyword co-occurrence network. The words were divided into four clusters with different colors: clinical characterization (red), basic research (green), clinical management of tooth agenesis (blue), and syndromic tooth agenesis (orange). The size of the node reflected the frequency of occurrence. (B) Visualization of the average time of keyword occurrence. Lighter color indicates a later appearance.

Keyword co-occurrence network could be used to describe the status quo of the knowledge map and frontier topics. A total of 6,477 keywords within the titles and abstracts were identified by VOSviewer; 102 keywords occurring at least 30 times were further selected and visualized. The terms “hypodontia, tooth agenesis, children, prevalence, genetics, mutation, gene, expression, *PAX9*, *MSX1*, *AXIN2*, and mouse” were the most prevalent topics from 2001 to 2021. The keywords were grouped into four clusters (Figure 6A), with cluster 1 referring to clinical characterization and cluster 2 referring to basic research. Furthermore, using the VOSviewer keyword overlay map, we analyzed the keyword distribution in different periods (Figure 6B). We noticed that the basic research was conducted earlier in this field, with keywords such as *PAX9*, *MSX1*, and mutation in dark color. However, we also found that *WNT10a*, *AXIN2*, and gene variants were colored yellow, suggesting newly identified risk genes in this field. Clinical characterization is seemingly a hot topic recently, with “prevalence” and “chemotherapy” having attracted more attention more recently.

Discussion

This study included 2,287 publications on tooth agenesis (2001 to 2021) from 2,469 institutions in 92 countries, with the participation of 9,803 authors, indicating that the field of tooth agenesis has attracted scholars’ attention worldwide. In this field, the USA was the biggest contributor. The University of Sao Paulo (Brazil) had the highest number of publications among the numerous research institutions worldwide. The University of Helsinki in Finland deserves special attention. Its publications were cited more than any other institution, indicating that the research results of this institution provide a unique reference value for the research in this field.

The top three prolific authors were Alexandre R Vieira, Dong Han, and Hailan Feng. They focused on finding and validating the role of a gene in dental agenesis and elucidating the possible molecular mechanism from a genetic viewpoint. For example, in recent years, Dong Han identified five novel Msx1 heterozygous variants in multiple non-syndromic tooth agenesis in Chinese families, expanding the variant spectrum of isolated tooth agenesis and providing valuable information for genetic counseling [2]. Attention to these authors can help better grasp the research movement in this field.

Regarding journals, research results on dental agenesis were more inclined to be published in orthodontics-related journals, such as the American Journal of Orthodontics and Dentofacial Orthopedics and the European Journal of Orthodontics. This may be because patients with congenitally missing teeth often present with occlusal disorders and are often diagnosed, treated, and reported by orthodontists [17]. However, the top 10 most cited articles were mainly published in genetics-related journals such as the American Journal of Human Genetics and Development.

Keywords can quickly capture the distribution and evolution of hotspots in the research field of tooth agenesis. A total of 102 keywords occurring at least 30 times were grouped into four clusters. The largest cluster was related to the clinical characterization of tooth agenesis. “Prevalence” was the most frequent keyword in this cluster. Prevalence of tooth agenesis has been reported worldwide; however, prevalence varies widely in published studies, depending on the population studied [7]. At the same time, there are differences in different ethnicities regarding the predilection sites of congenitally missing permanent teeth (third molars are excluded) [18-20]. It is worth mentioning that chemotherapy appeared in the cluster as a keyword and appeared later. Recent publications have shown a link between tooth absence and cancer [21]. Since Lammi et al. found that AXIN mutation can cause tooth agenesis and simultaneously cause colorectal cancer, subsequent studies have reported that patients with congenital tooth absence may have a higher cancer risk, possibly due to an overlap in the genetic basis for the occurrence of tooth agenesis and some specific cancers [10,22-24]. Focusing on the association of tooth agenesis with specific cancers and providing references for early diagnosis and treatment may be one of the trends for future research.

The second largest cluster was related to basic research of tooth agenesis. Benefiting from the advances in genetics, scholars have identified the roles of some genes in dental agenesis. At present, *MSX1*, *PAX9*, *EDA*, and *AXIN2* are the most frequently reported genes related to dental agenesis [25,26]. According to the recent publications of the top 10 prolific authors, screening the mutation types of these genes is the current research hotspot. Professor Zhuan Bian from China believes that using recombinant proteins to improve the defects caused by gene mutations may be a promising treatment [27].

The third largest cluster was related to the clinical management of tooth agenesis. Multidisciplinary management to deal with tooth agenesis has been recognized by clinicians. It refers to the cooperation between pediatric dentistry, restorative dentistry, orthodontics, oral surgery, and general dentistry to carry out restorative management for patients with a congenital absence of teeth to maintain and restore patients’ function and appearance [28]. Team members from the University of Newcastle upon Tyne in England published a series of articles in the British Dental Journal on the role of each discipline in promoting restorative treatment for patients with tooth agenesis [28-31]. Clinicians should master this concept.

The fourth cluster was associated with syndromic tooth agenesis. The primary keywords included “cleft palate,” “oral clefts,” and “orofacial clefts.” Teeth grow and develop with support from the craniomaxillofacial region, and lesions at this site severely affect odontogenesis. Compared with the general population, patients with cleft lip and palate and their unaffected brothers and sisters are more likely to have dental agenesis [32]. In addition, tooth agenesis could be a localized manifestation of a syndromic disease. At present, more than 150 syndromes have been reported to be associated with tooth agenesis, such as hypohidrotic ectodermal dysplasia, incontinentia pigmenti, Witkop tooth-nail syndrome, and Schope-Schulz-Passarge syndrome [27].

Some limitations in this study need to be noted. First, all the publications were screened from a single database (WOSCC), which might have led to selection bias. Second, this study only included publications in English, possibly ignoring high-quality research in other languages. Finally, the analysis and prediction of research hotspots in this study may not be completely accurate because only publications published before January 1, 2021, were included.

## Conclusions

The global research trends of tooth agenesis from 2001 to 2021 were summarized for the first time through bibliometric analysis. The USA and many countries have made important contributions to this field. The relevant research institutions in these countries have maintained close cooperative relations. Moreover, a large number of studies on tooth agenesis have been published in orthodontics-related journals. The findings could help clinicians and researchers identify potential collaborators and stay updated on the latest trends and developments. Multidisciplinary combination therapy for tooth agenesis has become a consensus that can guide clinical practice. For future prospects, our study suggests that genetic mutations related to tooth agenesis and the relationship between tooth agenesis and tumors are emerging areas of interest. Future research could focus on these topics to further our understanding of tooth agenesis and improve patient care. Additionally, collaborations between experts in genetics, dentistry, and oncology could be beneficial in advancing this field.
